# Quantitative trait variation is revealed in a novel hypomethylated population of woodland strawberry (*Fragaria vesca*)

**DOI:** 10.1186/s12870-016-0936-8

**Published:** 2016-11-04

**Authors:** Jihua Xu, Karen K. Tanino, Kyla N. Horner, Stephen J. Robinson

**Affiliations:** 1 Department of Plant Sciences, University of Saskatchewan, 51 Campus Drive, Saskatoon, Saskatchewan Canada; 2 Agriculture and Agri-Food Canada, Saskatoon Research Centre, 107 Science Place, Saskatoon, Saskatchewan Canada

**Keywords:** Epigenetics, DNA methylation, *Fragaria vesca*, Strawberry, 5-azacytidine, Quantitative trait variation

## Abstract

**Background:**

Phenotypic variation is determined by a combination of genotype, environment and their interactions. The realization that allelic diversity can be both genetic and epigenetic allows the environmental component to be further separated. Partitioning phenotypic variation observed among inbred lines with an altered epigenome can allow the epigenetic component controlling quantitative traits to be estimated. To assess the contribution of epialleles on phenotypic variation and determine the fidelity with which epialleles are inherited, we have developed a novel hypomethylated population of strawberry (2n = 2x = 14) using 5-azacytidine from which individuals with altered phenotypes can be identified, selected and characterized.

**Results:**

The hypomethylated population was generated using an inbred strawberry population in the *F. vesca* ssp. *vesca* accession Hawaii 4. Analysis of whole genome sequence data from control and hypomethylated lines indicate that 5-azacytidine exposure does not increase SNP above background levels. The populations contained only Hawaii 4 alleles, removing introgression of alternate *F. vesca* alleles as a potential source of variation. Although genome sequencing and genetic marker data are unable to rule out 5-azacytidine induced chromosomal rearrangements as a potential source of the trait variation observed, none were detected in our survey. Quantitative trait variation focusing on flowering time and rosette diameter was scored in control and treated populations where expanded levels of variation were observed among the hypomethylated lines. Methylation sensitive molecular markers indicated that 5-azacytidine induced alterations in DNA methylation patterns and inheritance of methylation patterns were confirmed by bisulfite sequencing of targeted regions. It is possible that methylation polymorphisms might underlie or have induced genetic changes underlying the observable differences in quantitative phenotypes.

**Conclusions:**

This population developed in a uniform genetic background provides a resource for the discovery of new variation controlling quantitative traits. Genome sequence analysis indicates that 5-azacytidine did not induce point mutations and the induced variation is largely restricted to DNA methylation. Using this resource, we have identified new variation and demonstrated the inheritance of both variant trait and methylation patterns. Although direct associations remain to be determined, these data suggest epigenetic variation might be subject to selection.

**Electronic supplementary material:**

The online version of this article (doi:10.1186/s12870-016-0936-8) contains supplementary material, which is available to authorized users.

## Background

Phenotypic variation is determined by a combination of genotype, environment and their interactions [1]. Through the use of carefully designed crossing strategies, pedigree analysis and molecular markers, the proportion of the phenotypic variation controlled by genetic components can be estimated and mapped to specific loci with the remaining variation attributed to environmental components [2–4]. Measuring the phenotypic variation observed among genetically uniform individuals allows the extent to which the environmental components affect quantitative traits to be estimated. Populations of inbred lines, the F_1_ generation derived from crossing inbred lines and the progeny from double-haploid individuals contain genetically identical individuals and are often used to estimate environmental variance [5–8]. However, it is becoming increasingly accepted that epigenetic polymorphism affects quantitative trait variation [9–11]. Epigenetic variation results from the same sequence allele possessing a different chromatin organization, modifying its propensity for expression or chromosomal interactions [12, 13]. Epigenetic variation has the potential to alter the magnitude of gene expression whereas genetic polymorphisms result from differences in the primary DNA sequence resulting in functional allelic variants. Establishing the effect that epigenetic variation has on quantitative characters is complicated by any underlying genetic variation [14] and it is often considered a component of environmental variation [13, 15, 16]. However, the development of tools that are able to detect and measure epigenetic variation in genetically uniform populations is allowing these issues to be resolved [9, 17].

Epigenetic information is stored in three molecular systems, namely, DNA methylation, post-translational modification of histone proteins and non-coding RNA molecules. Together, these systems organize the structure and configuration of chromatin adjusting its accessibility to the transcriptional machinery that can result in the activation or repression of gene expression [18, 19]. Perhaps the best studied of these systems is DNA methylation [10, 20]. In eukaryotes, DNA can be modified through the covalent attachment of a methyl group to the carbon atom at position five of the cytosine ring, a reaction catalyzed by methyltransferases [21, 22]. In contrast to animals, where cytosine methylation is largely restricted to the CG sequence context, DNA methylation in plants is additionally observed at positions with CHG and CHH sequence contexts (where H = A, C, T) [23]. DNA methylation status is maintained through DNA replication through the action of DNA maintenance enzymes such as DNA METHYLTRANSFERASE 1 (MET1) and CHROMOMETHYLASE 3 (CMT3) transferring the methylation status to the newly synthesized stand using symmetry in the CG and CHG sequence contexts [24, 25]. Whereas the action of *de novo* methylation enzymes including DOMAINS REARRANGED METHYLTRANSFERASE 2 (DRM2) are required to maintain the information at asymmetric sites CHH where siRNA molecules act as guides providing the necessary sequence specificity [26, 27]. Major differences in cytosine methylation patterns throughout plant genomes distinguish gene-rich regions from repeat-rich regions but variation in methylation among genes and their promoters has been shown to control expression [28]. Altering the constraints provided by DNA methylation to gene expression might manifest as changes in phenotypes and offers a mechanism for epigenetic control over quantitative traits. Additionally, alterations in DNA methylation patterns have the potential to be indirectly mutagenic, affecting the higher-order chromosome conformation leading to chromosomal rearrangements that might also lead to trait variation [29–32].

Although comparatively rare, examples of naturally occurring heritable traits controlled by epialleles are known. This is best exemplified with the demonstration that changes in DNA methylation patterns are responsible for the non-Mendelian inheritance of floral symmetry observed in Toadflax. These epigenetic alterations cause silencing of *Lcyc* gene expression, resulting in the easily observable change from bilateral to radial floral symmetry [33]. Additionally, fruit ripening in tomato was inhibited by a spontaneous epimutation resulting from hypermethylation of the SBP-box transcription factor promoter sequence [34]. Transgenerational fidelity of epigenetic information in plants has been demonstrated more generally and there is a growing body of evidence describing the importance of these phenomena [35, 36]. Stable inheritance of naturally occurring epialleles was demonstrated through genome-wide analyses in studies using *Arabidopsis* where the majority of DNA methylation patterns were faithfully transmitted through meiosis for many generations, dispelling the idea that these marks were largely transitory and subject to environmental change [37]. Stable inheritance of phenotypic variation in flowering time and plant height induced by altering DNA methylation patterns was observed for over eight generations in *Arabidopsis* using a population of epigenetic Recombinant Inbred Lines (epiRILs) suggesting that quantitative trait variation might also be under epigenetic control [9]. The variation among the epiRILs was generated in isogenic backgrounds through the functional inactivation of the maintenance methyltransferase resulting in lines with reduced DNA methylation primarily at CG sites [9].

Established patterns of DNA methylation can be stochastically changed through the application of potent pharmacological agents such as 5-azacytidine (5-azaC) [38, 39]. This compound is an analogue of cytidine possessing a nitrogen atom rather than a carbon atom at the 5-position of the pyrimidine ring and thus lacking the ability to form a bond with a methyl group. 5-azaC is metabolized to 5-aza-2′-deoxycytidine-triphosphate where it competes with cytosine as a substrate for DNA polymerase during DNA replication and is incorporated into the genome resulting in demethylation as marks cannot be transferred by maintenance methylase enzymes to the newly synthesized DNA strand [40, 41]. Studies altering genome-wide DNA methylation patterns have demonstrated that exposure to exogenous 5-azaC in plants can increase phenotypic trait variation. Strategies exploiting this phenomenon have been applied in a range of plant species where it has been demonstrated that dwarfism in *Oryza sativa* [42, 43], early flowering in *Arabidopsis* [44], *Linum usitatissimum* [45–47], and *Solanum ruiz-lealii* [48], as well as abnormal flower development, and leaf morphology *Solanum ruiz-lealii* [48, 49] have been identified.

The Rosaceae family contains the majority of the most economically important temperate fruit crops such as apple, cherry, pears, plum, peach, raspberry, almonds as well as strawberry [50]. Woodland strawberry (*F. vesca*, 2n = 2x = 14) has a rapid generation time, small plant stature, is able to reproduce through both sexual and clonal pathways and has a relatively small genome (~240 Mb). The major advantage that *F. vesca* offers for functional genomics is the availability of the genome sequence generated using the inbred line Hawaii 4 of the *F. vesca* ssp. *vesca* [51]. Together, these features have transformed this species into the model species for both strawberry and the wider Rosaceae family. The generation of the genome sequence paves the way for genomics analyses to determine the function of strawberry genes and is a prerequisite for detailed investigations describing epigenetic variation.

Here we describe the generation of a new resource that can be used in future analyses to address fundamental questions of epigenetic gene regulation and its contribution to quantitative phenotypic variation in *F. vesca.* This novel resource was developed using the Hawaii 4 genetic background with the prospect of generating and discovering novel factors (alleles or epialleles) that affect trait variation. We assess the extent of observed changes in DNA methylation patterns and phenotypic variation in these plants and assess whether this variation is transmitted through meiosis.

## Methods

### Plant materials

The *F. vesca* ssp. *vesca* accession Hawaii 4 (germplasm accession: PI551572) was used in this study and seeds obtained after seven generations of inbreeding (H4S7) derived through single-seed descent were kindly provided by Dr. Janet Slovin (USDA). These seeds were direct descendants of the individual plant used to generate the *F. vesca* reference genome (H4S4). The plant material used for population development was derived from seeds generated from an additional generation of inbreeding (H4S8) required to produce sufficient material for mutagenesis.

### Exposure of *F. vesca* to 5-azacytidine

A population of H4S8 seeds was treated with 5-azaC (Sigma-Aldrich). Seeds were imbibed with water for 24 h and then treated with 0, 1.0, 5.0, 20.0, 50.0 or 100.0 mM of 5-azaC and incubated at room temperature in the dark for six days before being rinsed and germinated on filter paper in petri dishes. Germinated seedlings were transferred to pots containing Sunshine Mix #4 (Sun Gro Horticulture), and placed on benches under greenhouse conditions where they were grown at 23 ± 2 °C day and 18 ± 2 °C night under an 18/6 h day/night photoperiod. Natural light was supplemented with 400 W high-pressure sodium lights at 600 μmol m^−2^ s^−1^. Plants were fertilized once per week with 2 g/L of NPK (20-20-20) including micronutrients (Plant Products Co. Ltd.).

### Phenotypic traits assessments

The 5-azaC treated and control plants were assigned a code as a unique accession identifier (ERFv#) to ensure phenotypic assessment was conducted without bias. The 5-azaC treated and control populations were scored for two phenotypic traits: (1) Flowering time, recorded as the number of days from sowing to anthesis, determined by the opening of the first (primary) flower; (2) Rosette diameter (mm), recorded as the maximal linear distance across the strawberry rosette 45 days after sowing.

### Preparation of strawberry genomic DNA

Fresh leaf material was harvested directly into liquid nitrogen and stored at −80 °C until DNA extraction. DNA from ~400 mg of leaf material was extracted using the CTAB (cetyltrimethyl ammonium bromide) method [52] with the following modifications. To obtain high-quality DNA from strawberry, the tissue was ground in liquid nitrogen to a fine powder and transferred to a sorbitol buffer (100 mM pH 8.0 Tris–HCl, 0.35 M sorbitol, 5 mM pH 8.0 EDTA, 1 % PVP-40 with 1 % 2-mercaptoethanol) which was used as a wash buffer to remove excessive mucilaginous polysaccharides prior to CTAB extraction [53]. DNA quantification was performed using Qubit 2.0 Fluorometer and the Qubit dsDNA BR Assay Kits (Invitrogen) according to the manufactures instructions.

### Assessment of genetic variation using Amplified Fragment Length Polymorphism (AFLP)

The AFLP protocol described by [54] was followed with modifications: A total of 250 ng of genomic DNA extracted from leaf material was digested with 10 units *Eco*RI, 5 units *Mse*I (New England Biolabs), in NEB-4 buffer with BSA in a final volume of 40 μl for 2 h at 37 °C and the enzymes denatured by incubation for 15 min at 70 °C. Ligation of adaptors (Additional file 1: Table S1a) to the *Eco*RI and *Mse*I digested DNA was performed using NEB-4 buffer, BSA, ATP and 100 cohesive end units of T4 DNA ligase (New England Biolabs) in a total volume of 50 μl at room temperature for 2 h. The ligation reaction was diluted 1:10 before AFLP pre-selective amplification. Pre-selective PCR reactions was performed in a volume of 50 μl containing 5 μl of 1:10 ligation dilution, 0.1 μM of the *Eco*RI and *Mse*I primers (Additional file 1: Table S1a), 1× PCR buffer with MgCL2, 200 μM dNTP and 1 unit of *Taq* polymerase. The conditions for pre-selective PCR were as follows: 19 cycles of 94 °C for 30 s, 56 °C for 1 min, and 72 °C for 1 min.

The product of pre-selective amplification was diluted 1:50 and used as template in the selective amplification reaction. Selective amplification reactions were performed in a final volume of 25 μl containing 5 μl of the diluted pre-selective amplification product, 0.05 μM ^33^P labeled *Eco*RI selective primer (Additional file 1: Table S1a), 0.25 μM *Mse*I selective primer (Additional file 1: Table S1a), 1× PCR buffer, 200 μM dNTP and 1 unit of *Taq* polymerase. The conditions for selective PCR were as follows: 12 cycles of 94 °C for 30 s, 65 °C for 30s, and 72 °C for 1 min, then followed by 22 cycles of 94 °C for 30 s, 56 °C for 30s, and 72 °C for 1 min.

### DNA sequencing libraries construction

Whole genome DNA sequencing was conducted in three control lines and four 5-azaC treated lines. Illumina TruSeq DNA libraries were prepared following the manufactures’ instructions. Briefly, one μg of whole genomic DNA was sheared using the Bioruptor (Diagenode) using 12 cycles, pulsing for 30 s with 190 s gap between pulses. Following fragmentation, end repair, and adapter ligation, the BluePippin Prep (Sage Science) was used to capture 590 bp fragments. The libraries were quantified using the 2100 Bioanalyzer (Agilent Technologies) and sequencing was performed using Illumina HiSeq 2000 platform according to the manufacturer’s instructions.

### Sequence alignment, Single Nucleotide Polymorphism (SNP) identification, and SNP annotation

Sequence reads in fastq format were filtered and trimmed using Trimmomatic v0.32 [55]. Sequence quality assessment was conducted using CLC Genomic Workbench 8.5. The filtered libraries were aligned to the reference *F. vesca* whole genome (v1.0) [51] using CLC Genomic Workbench 8.5. Variant calling was performed using HaplotypeCaller and SNP identification was performed using SelectVariants with Genome Analysis Toolkit (GATK) [56]. Further filtering of SNP variant calls was performed using custom Perl scripts where high confidence SNP were identified by selecting for those loci with at least three reads, that were not adjacent to an identified Indel (adjacency was determined by the length of the detected Indel) and did not share a common genotype in control and 5-azaC treated samples. SNP annotation and functional prediction of the variants were performed using SnpEff [57] based on the annotations provided by the *F.vesca* genome v1.0 [51]. The protein sequences for the set of Arabidopsis flowering time genes listed at http://www.mpipz.mpg.de/14637/Arabidopsis_flowering_genes [58] obtained from TAIR were used to identify putative *F. vesca* flowering time homologues through sequence alignment.

### Assessment of DNA methylation polymorphism using Methylation Sensitive Amplified Polymorphisms (MSAP)

The MSAP protocol was followed with slight modification of the original protocol [59]. Briefly, genomic DNA from each sample analyzed was digested separately with 10 units *Eco*RI/5 units *Hpa*II (New England Biolabs) and 10 units *Eco*RI/10units *Msp*I (New England Biolabs). The *Eco*RI and *Hpa*II-*Msp*I adaptors (Additional file 1: Table S1b) were annealed and ligated to digested DNA fragments. The pre-selective and selective primers were listed in Additional file 1: Table S1b. Amplification of DNA fragments for MSAP followed the same PCR cycling conditions used for AFLP.

### Resolution and scoring of amplified AFLP and MSAP products

The selective PCR amplification products from AFLP and MSAP were resolved using a 5 % polyacrylamide gel using the BioRAD Sequi-Gen vertical polyacrylamide gel system. The resulting gel was dried and exposed to autoradiographic film (Kodak BioMax MR film 35 × 43 cm). The size of the visible fragments was determined using ^33^P labeled 50 bp ladder. A total of four and ten primer pairs were used to assay for polymorphism using the AFLP and MSAP method respectively (Additional file 1: Table S1).

Scoring of the AFLP and MSAP data were restricted to the clearly amplified fragments and data were recorded as dominant allelic markers. The banding patterns representing each observed allele in each individual were encoded by single and double band values for the AFLP and MSAP data respectively. In Additional file 2: Figure S1 for each locus, if there were bands resolved after electrophoresis in both *Eco*RI/*Hpa*II and *Eco*RI/*Hpa*II digest lanes, it was scored as 1/1 (type I band). In this situation, cytosine was not methylated. When the bands were present in the *Eco*RI/*Hpa*II digest and absent in the *Eco*RI/*Hpa*II digest it indicated that cytosine methylation was present on one strand of the DNA, called hemimethylation and scored as 1/0 (type II band). Although hemimethylation can occur in both external and internal cytosines or only in the external cytosine of the 5′-CCGG-3′ recognition sequence, the former pattern is of higher frequency. When the bands were present in the *Eco*RI/*Hpa*II digest and absent in *Eco*RI/*Hpa*II digest, the internal cytosine methylation in both strands was methylated and scored as 0/1 (type III band). If there were no bands, it was scored as 0/0 (type IV band) showing fully methylation, and both internal and external cytosine methylation patterns having a higher frequency compared to only external cytosine methylation form.

### Generation of high-resolution DNA methylation patterns at target loci

Putative CpG islands in the *F. vesca* genome were identified using a custom Perl Script. CpG islands were defined as being a minimum of 300 bp with greater than 50 % GC content and an observed-CG/expected-CG ratio greater than 0.6. Three target regions were selected using the *F. vesca* genome v1.0 to determine the methylation patterns that are enriched for cytosine bases. Target region one is on chromosome one between positions 1107633 and 11077319; Target region two is on chromosome two between positions 1029956 and 1030513 and Target region three is on chromosome four between positions 4884809 end 4885267. To ensure efficient amplification PCR primers were designed to amplify products less than 500 bp since conversion using sodium bisulfite can degrade the integrity of genomic DNA [60]. To ensure efficient annealing, primers were designed to avoid the presence of cytosine bases making them able to amplify from sequences possessing either methylated or unmethylated cytosine bases. The primer sequences used are presented in Additional file 3: Table S2.

Genomic DNA was treated with sodium bisulfite using the EZ DNA Methylation-Gold Kit (Zymo Research), by incubation at 98 °C for 10 min, 64 °C for 2.5 h in a thermal cycler. Lambda DNA (150 ng) was spiked into each sample as an unmethylated reference to calculate conversion rate efficiency. The converted DNA was used as template DNA in the PCR to amplify target genomic loci. The PCR was performed in 50 μl final volume with Zymo*Taq* ^TM^ Premix 25 μl (Zymo Research), 5 μl of each primer (10 μM), template DNA and H_2_O 15 μl. The conditions for PCR were as follows: 95 °C for 10 min followed by 40 cycles of 95 °C for 30s, 55 °C for 40s, and 72 °C for 60s with a final extension step at 72 °C for 7 min. The PCR product was sequenced and aligned to the reference sequence using Clustal Omega (http://www.ebi.ac.uk/Tools/msa/clustalo/). CyMATE was used for visualization to detect the methylation patterns (http:// cymate.org/cymate.html) [61].

### Statistical analysis

The quantitative phenotypic data obtained from measuring flowering time and rosette diameter were analyzed using the statistical software R [62]. Basic descriptive statistics including the mean and variance were estimated from the control population for each character, and the significance of each deviation from the control population mean was determined using one sample Z-test.

MSAP profiles describing the methylation patterns observed in the 5-azaC treated and control lines were summarized by Principal Coordinates Analysis (PCoA) and compared by Analysis of Molecular Variance test (AMOVA) using the MSAP analysis package for R [63]. Loci with at least 5 % methylated levels were defined as methylation-susceptible loci. Polymorphic methylation-susceptible loci were defined when at least two individuals were non-methylated [64, 65].

## Results

### Generation of a hypomethylated population of *F. vesca*

Approximately 500 *F. vesca* seeds from generation H4S8 were exposed to a range of 5-azaC concentrations (0–100 mM). A total of 305 plants survived 5-azaC treatment and transplantation into soil. These were complemented with a population of 59 H4S8 control plants. The surviving treated population was composed of plants exposed to a range of 5-azaC concentrations (1.0, 5.0, 20.0 or 50.0 mM), whereas the control population was exposed to water. Seeds exposed to 5-azaC concentrations above 50 mM were unable to survive and the largest class, comprising ~40 % of the population, was exposed to 20 mM 5-azaC.

### Genetic uniformity was verified among the *F. vesca* populations

Since any genetic polymorphism is likely to complicate the effects resulting from induced epigenetic differences [66], in order to attribute any phenotypic variation observed in quantitative characters to epigenetic variation, it is necessary to perform the experiment using a genetically uniform population [14]. The Hawaii 4 lines used in this study were highly inbred, derived through single-seed descent for a total of eight generations (H4S8). This level of inbreeding strongly suggested the material used to develop the population was genetically uniform. This was initially confirmed using AFLP markers to assess the genetic background of the *F. vesca* material used in this study. Genotyping was performed using a randomly selected subpopulation consisting of five control lines and 22 lines from the hypomethylated population. Although AFLP markers are dominant, the large number of loci amplified per primer pair means they can be used to quickly survey the entire genome [67]. A total of 219 AFLP loci were amplified using four primer pair combinations (Additional file 1: Table S1a). The allelic banding patterns observed were identical throughout each of the 27 individuals examined, indicating that no introgression of alien *F. vesca* alleles had inadvertently occurred through hybridization during inbreeding (Additional file 4: Figure S2).

Further evidence of genetic uniformity was achieved by whole genome sequencing of selected lines to address the potential of 5-azaC to act as a mutagen. A total of seven lines, comprising three untreated and four 5-azaC treated lines were sequenced which resulted in the generation of 29,569,617 sequence reads. After exclusion of reads comprising low quality bases and trimming adaptors sequences, a total of 21,502,412 reads were aligned to the reference genome of *F. vesca*. The sequence alignments resulted in 187 MB (~90 %) coverage of the genome with the coverage depth ranging from 8 to 14 (Additional file 5: Table S3).

SNP loci were identifed from the short read sequence alignments for each of the seven lines using GATK. High confidence SNP detection required evidence for an alternate allele from at least three independent sequence reads, with a SNP quality score of >3000. Additionally, SNP loci were excluded when positioned adjacent to an identified Indel as these are likely to result from alignment artefacts. The total number of loci with high quality SNP was 30,685 where an alternate allele was present in at least one of the seven lines. However, for the vast majority of these loci (29,137 (95 %)), although the H4S8 allele differed from the reference allele, the genotype of all seven of the H4S8 lines was identical. The remaining 1548 (5 %) loci where variation among the seven lines was observed were further partitioned. A total of 1208 (4 %) loci were heterozygous and 340 (1 %) of the total loci possessed homozygous alternative alleles in at least one of 5-azaC treated lines and this genotype was absent from the control lines. Further inspection of the data revealed 153 (0.5 %) loci where greater than one alternative allele was detected among the seven lines, these might result from spontaneous mutation. Among these, both alternate alleles were found in the control and 5-azaC treated lines for 148 (97 %) of the 153 loci. At the remaining 5 (3 %) loci the alternate alleles were found exclusively among the 5-azaC treated individuals. The slight increase in allelic complexity (3 % increase in the loci possessing an extra allele) observed among the 5-azaC treated compared to control lines was not statistaically significant when testing for an increase in the propotion of loci with greater than two alleles (*χ* ^2^ = 0.0589; *p* = 0.8083). The biological significance of the detected SNP was assessed by assigning functional annotation to the SNP loci using SnpEff classified using the *F. vesca* genome annotation [51]. The majority 1365 (88 %) of the 1548 polymorphic loci identified were found in intergenic regions with only 183 (12 %) of the loci annotated as being in genes. Base changes resulting in the predicted loss of gene function accounted for 11 (0.7 %) of the loci, 119 (8 %) loci predicted nonsynonymous bases changes and 53 (3 %) loci resulted in prediction of synonymous base changes. No SNP were detected in *F. vesca* homologues of the flowering time genes in *Arabidopsis*. In the case of those loci with SNP variation found exclusively in the 5-azaC treated lines, six were predicted to result in loss of gene function, 46 nonsynonymous and 14 synonymous substitutions with 346 annotated in intergenic regions.

### Expanded phenotypic variation was observed in the 5-azaC treated population

**Fig. 1:**
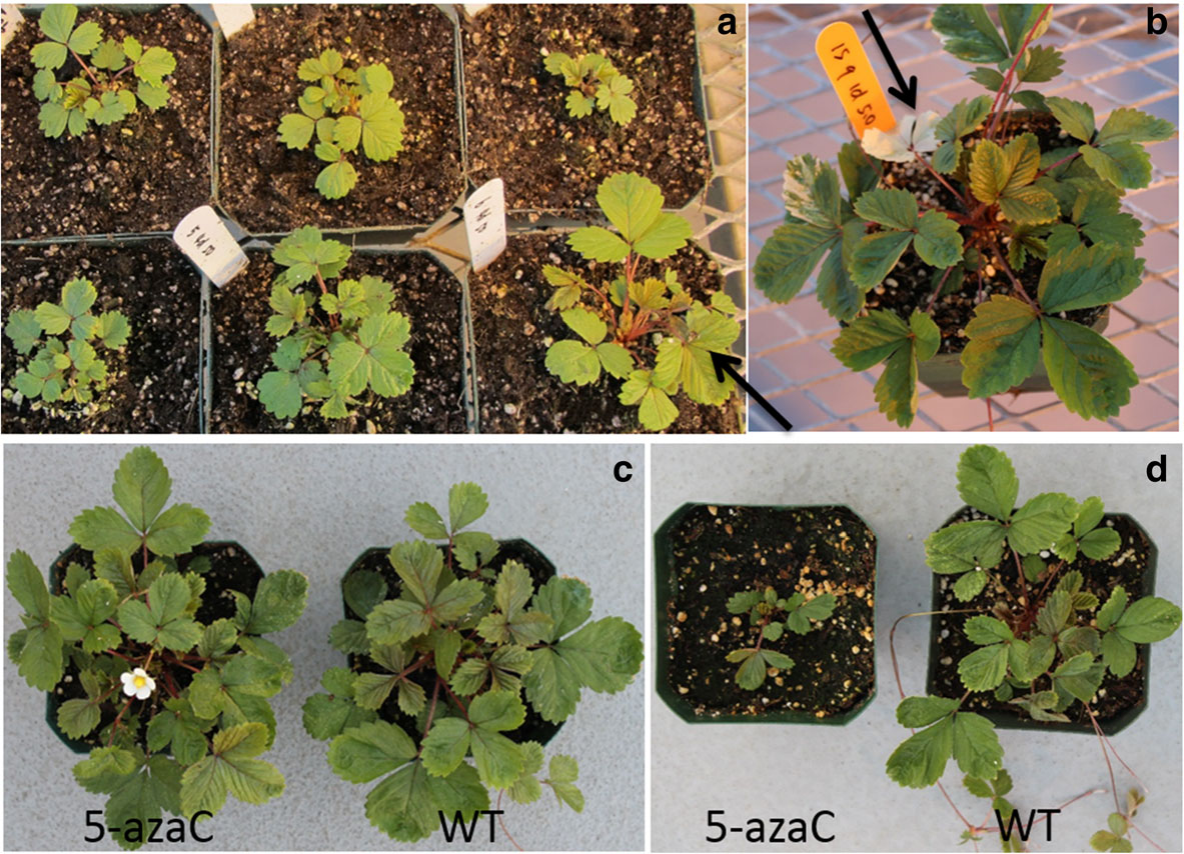
Phenotypic variation in plants subjected to 5-azaC treatments grown under greenhouse conditions. **a** An example of six individual plants varying in plant growth and development parameters under 50 mM 5-azaC treatment, right bottom plant ERFv 153 showed early flowering (see *arrow*); **b** Variegated chlorophyll pigmentation was observed in the progeny of one late flowering line ERFv 134 (see *arrow*); **c** The comparison of flowering time between wild type (*right*) and 5-azaC treatment line (*left*); **d** The comparison of plant rosette diameter and stature between wild type (*right*) and 5-azaC treatment line (left)

**Fig. 2:**
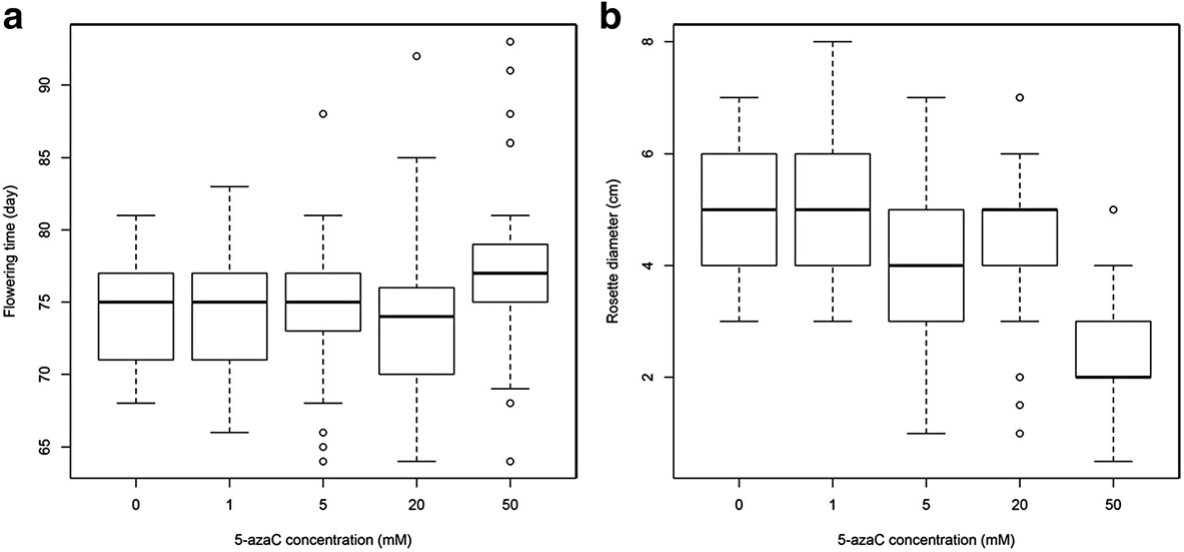
Distribution of quantitative phenotypes for control and epimutagenized lines. **a** Phenotypic variation observed for flowering time; **b** Phenotypic variation observed for rosette diameter

A number of phenotypic differences were observed upon visual examination of the individuals comprising the 5-azaC population. Quantitative characters including flowering time and plant rosette diameter were scored (Fig. 1). These data were summarized using descriptive statistics and the distributions were visualized for each subpopulation of plants, exposed to different concentrations of 5-azaC (Fig. 2). Individuals selected differed significantly from the mean of the control phenotypic values for flowering time and rosette diameter. The population distribution for each phenotype was summarized (Fig. 2a) where the distributions indicate the effect of 5-azaC treatment resulted in expanded variation for these quantitative traits. Treatment with low concentrations of 5-azaC (1.0 mM and 5.0 mM) caused little deviation from those observed among the control lines for flowering time. Treatment with higher levels of the 5-azaC (20 mM and 50 mM) was required to induce a wide range of phenotypic variation that was observed at both tails of the distribution (Fig. 2a). The treatment did not appreciably alter the central values of the distributions, where the median flowering time of 20.0 mM treatment was one day earlier than the control while the average flowering time for 50.0 mM was three days later. As anticipated, the greatest variation was observed among those plants exposed to the highest 5-azaC concentrations. Similar to the data collected describing flowering time, exposure to 5-azaC increased the variance for rosette diameter. Rosette diameter appeared to be more susceptible to alterations induced by 5-azaC as these lines tended to be smaller than the diameters measured among the control lines (Fig. 2b). This was particularly evident among those plants exposed to the higher concentrations (20 mM, 50 mM) of 5-azaC. The rosette diameters of those plants exposed to 50 mM 5-azaC were the smallest with the distribution skewed towards smaller diameters, rather than possessing outliers at both tails of the distribution as observed for flowering time.

**Fig. 3:**
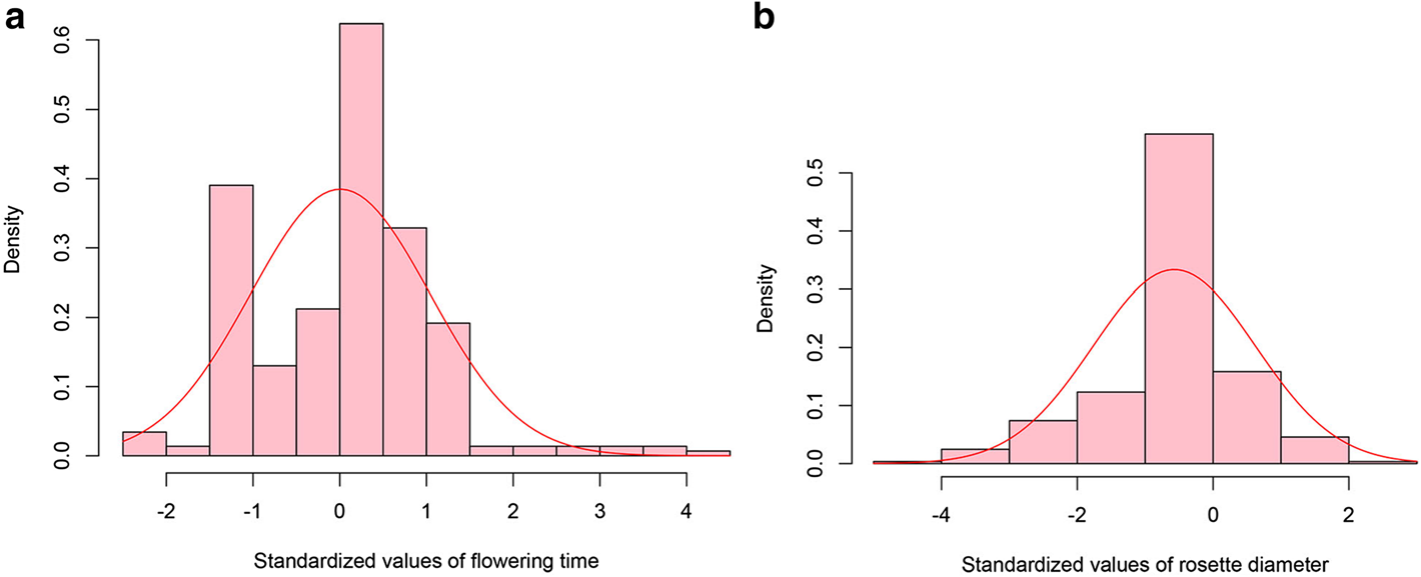
Distribution of standardized values for two quantitative traits observed in the epimutangenized population relative to the control population. **a** Flowering time density histogram of Z-test values from 292 hypomethylated lines; **b** Rosette diameter density histogram of Z-test values from 284 hypomethylated lines

Ranking of phenotypic values identified those individuals with the most extreme phenotype. There were a total of five (1.7 %), nine (3.1 %), one (0.3 %) and 28 (9.8 %) individuals identified that had a significantly different (*p* < 0.05) early flowering time, late flowering time, large diameter or small diameter respectively (Fig. 3, Table 1). The majority of these variant phenotypes were observed among the lines exposed to higher concentrations of 5-azaC (Table 1).

**Table 1:** Distribution of variant phenotypes among 5-azaC concentration classes

	1.0 mM	5.0 mM	20 mM	50 mM
Early flowering lines	0	2	2	1
Late flowering lines	1	2	1	5
Large diameter lines	1	0	0	0
Small diameter lines	0	9	4	15

### Alteration in DNA methylation pattern was detected in the 5-azacytidine treated population

**Fig. 4:**
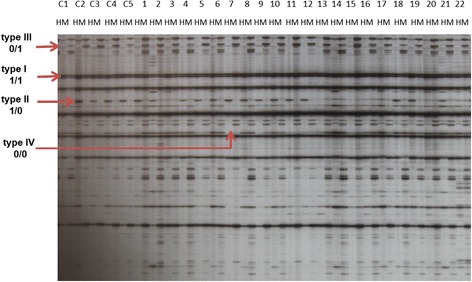
Example of MSAP profiles and classification of four types of bands. MSAP banding pattern obtained using the primer combination *Eco*RI + ACA and *Hpa*II/*Msp*I + TAA C1-C5: Control lines. 1–22: Randomly selected hypomethylated lines. H: DNA subjected to *Eco*RI / *Hpa*II digestion. M: DNA subjected to *Eco*RI / *Msp*I digestion. The arrows indicate type I, type II, type III and type IV bands amplified. The “1” represents the presence of bands and “0” represents the absence of bands for scoring purposes

Exposure to 5-azaC is known to cause alterations in DNA methylation. To assess the efficacy of the drug we surveyed the same subpopulation used for the AFLP analysis, assaying for variation in cytosine methylation status using the MSAP protocol. A total of 246 MSAP loci were amplified from each individual using ten primer pair combinations. In contrast to the AFLP profiles observed, the MSAP banding patterns revealed a range of polymorphic loci. The MSAP patterns reveal distinct cytosine methylation status at the sampled CCGG restriction sites (Fig. 4). These patterns were grouped into four classes allowing estimates of DNA methylation, at sampled sites to be generated (Table 2). As anticipated, the level of DNA methylation observed was negatively correlated to the concentration of 5-azaC to which the plants were exposed (Table 2). These data indicate 5-azaC reduced DNA methylation within the treated individuals. Although a significant reduction in DNA methylation was observed upon exposure to 5-azaC when compared to controls (Additional file 6: Table S4), the major contribution to this reduction (*P* < 0.05) occurred when the concentration was increased to 50 mM (Table 2). The MSAP data revealed that changes induced by 5-azaC are the most prevalent in the type I (unmethylated class) and type IV (fully methylated class). Exposure to 5-azaC increases the frequency of loci that possess no methylated cytosine (45 to 52 %) and decreases the frequency of CCGG sites with four methylated cytosine bases (21 to 14 %). The frequency of the type II MSAP banding pattern (hemimethylated) showed no change and the type III (internal base methylation) showed a minor reduction in frequency. Examination of the methylated cytosine bases (type II + type III + type IV) in each CCGG site in the control sample revealed that the majority (54 %) occurred at a single cytosine base on each strand and 38 % were observed with methyl groups on all four cytosine bases. The remainder (8 %) of the methylation was found in a hemimethylated context.

**Table 2:** Summary of DNA methylation profiles observed in individuals exposed to different concreations of 5-azaC

Treatment (mM)	Type I (1/1)	Type II (1/0)	Type III (0/1)	Type IV (0/0)	Methylated cytosine %
0	110	11	73	52	38.37
1.0	112	11	78	46	36.67
5.0	114	9	74	51	37.2
20.0	117	8	77	44	35.04
50.0	129	11	70	35	30.77

Methylated cytosine (%) = [(II*2 + III*2 + IV*4) / ((I + II + III + IV)*4)]* 100

A total of 246 amplified loci were scored in every individual

**Fig. 5:**
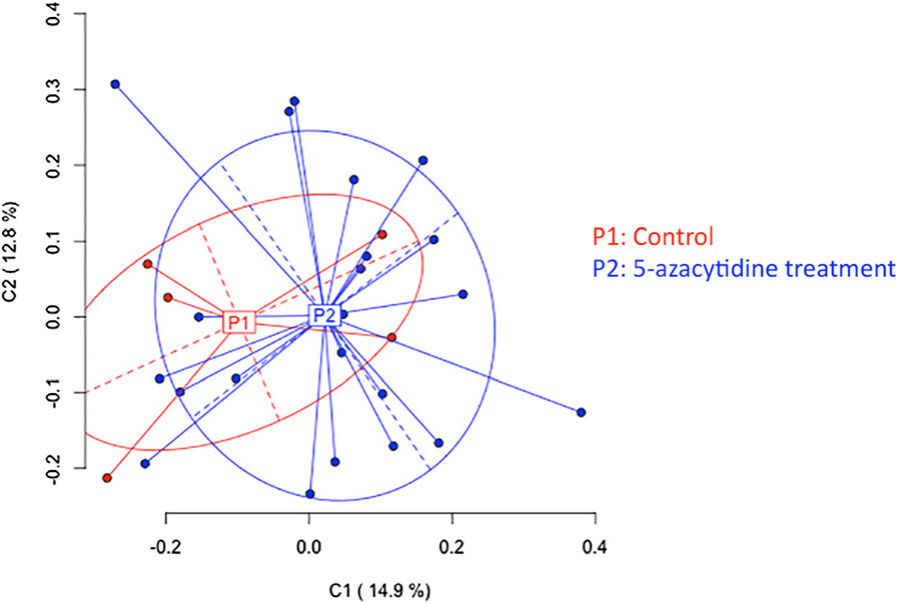
Principal Coordinates Analysis (PCoA) for DNA methylation differentiation between control lines and 5-azaC treatment lines using polymorphic methylation-susceptible loci (MSL) data. The percentages in the first two coordinates (C1 and C2) show the contribution of variance (shown in brackets). Color-labeled P1, P2 are the centroids of the respective group. P1 represents five control lines and P2 represents 22 hypomethylated lines

The DNA methylation patterns observed using MSAP among the 5-azaC treated population were summarized using PCoA. A total of 109 (44 %) MSAP loci passed filtering and were designated as methylation-susceptible loci, these loci were used in a PCoA and the variances of the two largest components were plotted to describe the variation in both the control and 5-azaC treated groups (Fig. 5). This analysis summarizes the highly dimensional MSAP data demonstrating that variation in DNA methylation patterns were observed among the sampled control lines and that the level of variation increased in the 5-azaC treated population. As anticipated, a large overlap was observed between the two populations, where the variation among the treated individuals expanded to occupy greater space encompassing the control lines, resulting in no significant difference between the two populations being detected by AMOVA (epigenetic distance Φst = −0.006, *P* = 0.52). The additional variation induced in the 5-azaC treated lines was revealed by a positive shift along the axis of both the first and second components, which explained 15 and 13 % of the variance respectively. The number of MSAP banding patterns observed in each of the 27 individuals at each of the 246 loci was summarized and used to estimate percent methylation at each of the sampled cytosine bases (Additional file 7: Table S5). The magnitude of DNA methylation change was correlated with 5-azaC concentrations, although variation among the control lines was observed. Examination of the strawberry hypomethylated population using MSAP markers indicated changes in DNA methylation up to 10 fold beyond the background with a 2 % range detected among control lines (37.2 to 39.8 %) whereas the variation among the hypomethylated population ranged by 20 % (19.1 to 39.2 %). Curiously, although the observed changes in the hypomethylated population indicated a general depletion of DNA methylation, there were individuals where DNA methylation increased. Interestingly, the line showing the greatest reduction in DNA methylation (ERFv 153) was also present in an early flowering phenotype (Fig. 1a).

### Inheritance of variant phenotypic traits

**Fig. 6:**
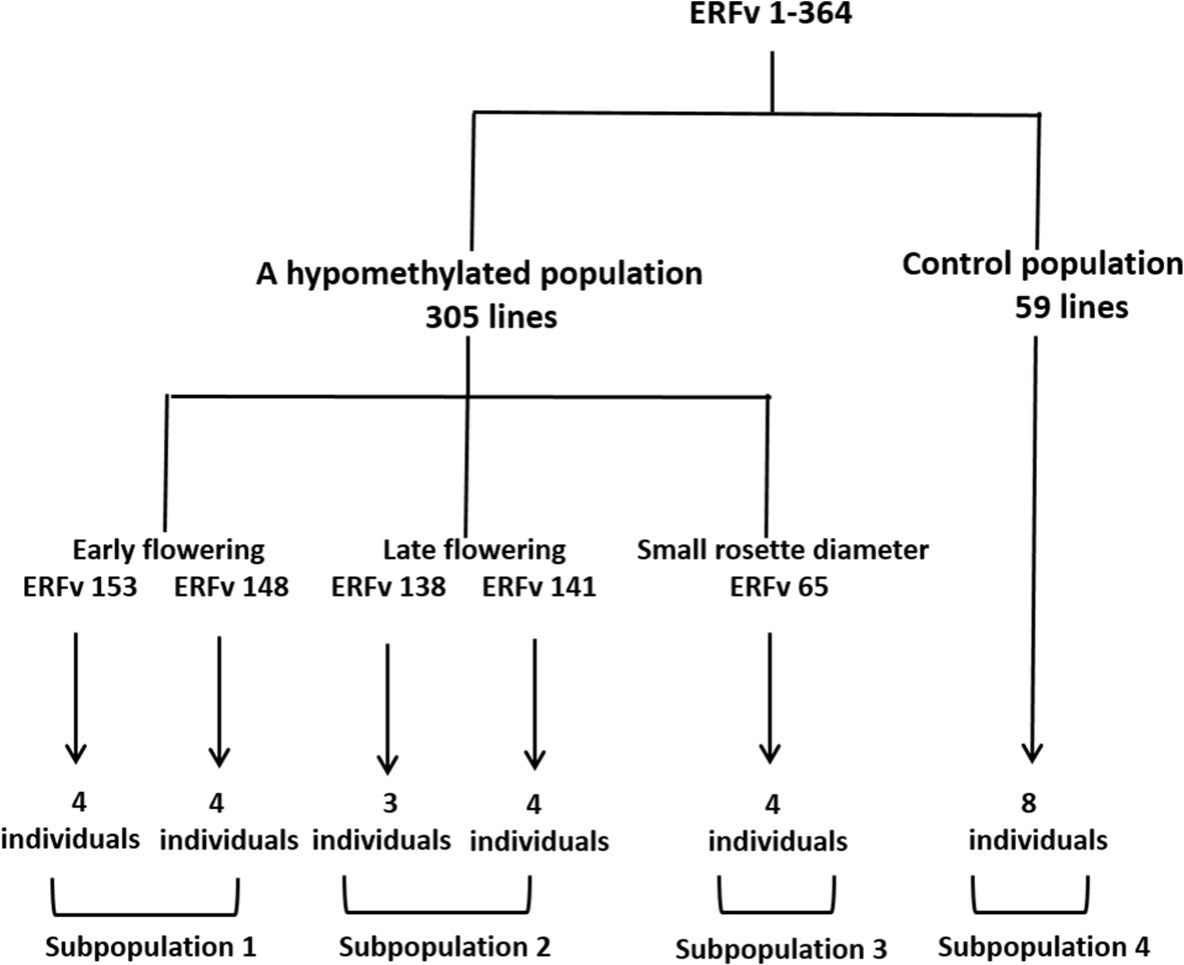
Overview of the hypomethylated population and subpopulation used in the MSAP study. A set of 364 lines was obtained including 59 control lines and 305 epimutagenized lines. Four subpopulations consisting of 8 individuals of early flowering, 7 individuals of late flowering, 4 individuals of small rosette diameter and 8 individuals of control lines from the next generation were used in the MSAP study

**Fig. 7:**
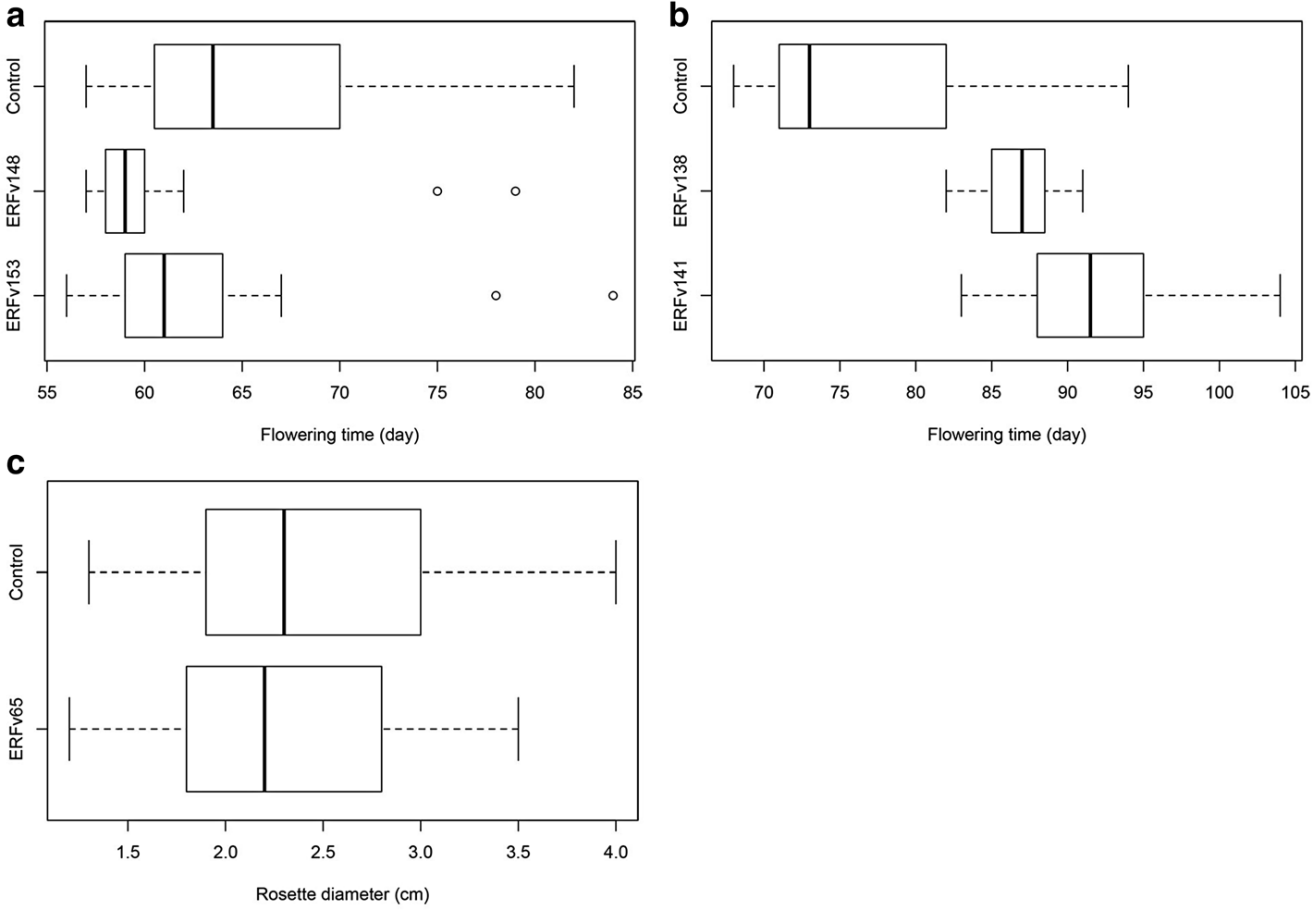
Distribution of flowering time and rosette diameter in the progeny of lines selected with variant traits. **a** Distribution of days to flowering among the progeny of control and the early flowering lines ERFv148 and ERFv153. The flowering time of the parental control, ERFv148 and ERFv153 lines were 75, 64 and 64 days respectedly. **b** Distribution of days to flowering among the progeny of control and the late flowering lines ERFv138 and ERFv141. The flowering time of the parental control, ERFv138 and ERFv141 lines were 74, 93 and 92 days, respectively. **c** Distribution of rosette diameter among the progeny of control and the dwarf line ERFv65

A total of five individuals were selected from the initial 5-azaC treated population (H4S8). The selected lines included the early flowering lines ERFv148 and ERFv153, the late flowering lines ERFv138 and ERFv141 and a line with a small rosette diameter ERFv65 (Fig. 6). Progeny (H4S9) from each of these individuals were grown where flowering time and rosette diameter were measured. Transmission of the variant flowering time phenotypes to their progeny was observed in each of these selected lines (Table 3 & Fig. 7). The average flowering time of the early flowering lines ERFv148 and ERFv153 progeny was four and three days earlier than control lines respectively. The progeny from the late flowering lines ERFv138 and ERFv141 had a greater difference being ten and 15 days later than the control lines respectively. However, the progeny from ERFv65 possessed an average rosette diameter 0.3 cm less than control lines and the distributions did not differ (Fig. 7). The extreme flowering time values observed in the H4S9 generation were found within treated families rather than within control families when the phenotypic scores were ranked. Although there was variation observed among the progeny, the family median values had shifted and were significantly different from control lines as demonstrated using the non-parametric Wilcoxon Rank sum test. This was best exemplified in the progeny from the late flowering line ERFv141 where the distributions were distinct and average flowering time had become significantly later (*P* = 1.951e-06) (Fig. 7).

**Table 3:** Descriptive statistics summarizing trait variation in the progeny of the selected lines

	Early flowering (day)			Late flowering (day)			Small rosette diameter (cm)	
	ERFv148	ERFv153	Control	ERFv138	ERFv141	Control	ERFv65	Control
Mean	60.6*	62.9	65.8	86.9*	91.7*	76.5	2.2	2.5
SD	5.6	7.2	6.9	3.0	6.5	7.1	0.7	0.7
Size	21	19	36	7	14	37	21	32
Min	57	57	57	82	83	68	1.2	1.4
Max	79	84	82	91	104	94	3.5	3.7

*p < 0.05

### Inheritance of DNA methylation patterns

**Fig. 8:**
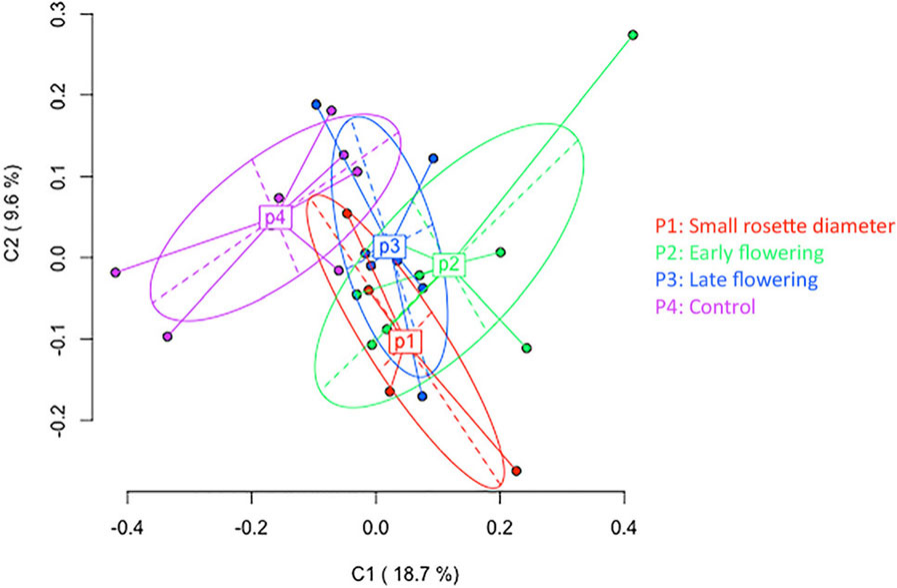
Principal Coordinates Analysis (PCoA) for DNA methylation differentiation between experimental groups using polymorphic methylation-susceptible loci (MSL) data. The percentages in the first two coordinates (C1 and C2) show the contribution of variance (brackets). Color-labeled P1, P2, P3, and P4 are the centroids of the respective group. P1: four small rosette diameter lines from line ERFv65. P2: four early flowering lines from line ERFv153, and four early flowering lines from line ERFv148. P3: three late flowering lines from line ERFv138, and four late flowering lines from line ERFv141. P4: eight control lines

The transmission of DNA methylation patterns through meiosis was assayed by subjecting the progeny with extreme phenotypes from each of the four phenotypic classes, small rosette diameter (P1); early flowering (P2); late flowering (P3); and control (P4) to phenotypic evaluation and MSAP analysis. The H4S9 generation consisted of 27 individuals comprised of four individuals from the P1 class, eight individuals from the P2 class, seven individuals from the P3 class, and eight individuals from the P4 class (Fig. 6). MSAP analysis of the individuals from the H4S9 generation yielded a total of 333 loci where 43 % were identified as methylation-susceptible loci. These data were subjected to multivariate analysis using PCoA to summarize the relationships among the individuals. The first two coordinates explained 28 % of the total variance in DNA methylation with the first coordinate explaining 19 % variance (Fig. 8). As expected, the plot of the P4 class (control lines, H4S9) displayed a similar pattern, occupying the same area and shape as the control lines examined in the previous generation (Fig. 5). The MSAP data for the individuals in each of the phenotypic classes (P1-3) clustered together and were separate from the control population. The largest differences were observed between those individuals exhibiting an early flowering phenotype (P2) and the control population (P4), where the distance (Φst = 0.1032, *P* < 0.0002) separating the clusters was found to be significant. Pairwise comparisons showed that the methylation patterns in classes P1, P2 and P3 were significantly different from the control class (P4) (Table 4). The most significant difference was detected between the control and the early flowering population (*P* < 0.0001) and this difference is visualized by the formation of two distinct clusters in Fig. 8. Interestingly, variegated chlorophyll pigmentation was observed in a single individual derived from the late flowering line ERFv134 (Fig. 1b), suggesting that novel phenotypes previously unobserved in the initial population might be revealed due to segregation of methylation patterns or other underlying factors in subsequent generations.

**Table 4:** DNA methylation differentiation between progenies derived from individuals with variant traits

Samples pairs		Φst between samples	*p* value
Control (P4)	Small plant diameter (P1)	0.1288	0.0190
Control (P4)	Early flowering (P2)	0.1869	0.0001
Control (P4)	Late flowering (P3)	0.1174	0.0010
Late flowering (P3)	Small plant diameter (P1)	0.0308	0.2204
Late flowering (P3)	Early flowering (P2)	0.0356	0.0674
Early flowering (P2)	Small plant diameter (P1)	0.0144	0.2950

**Fig. 9:**
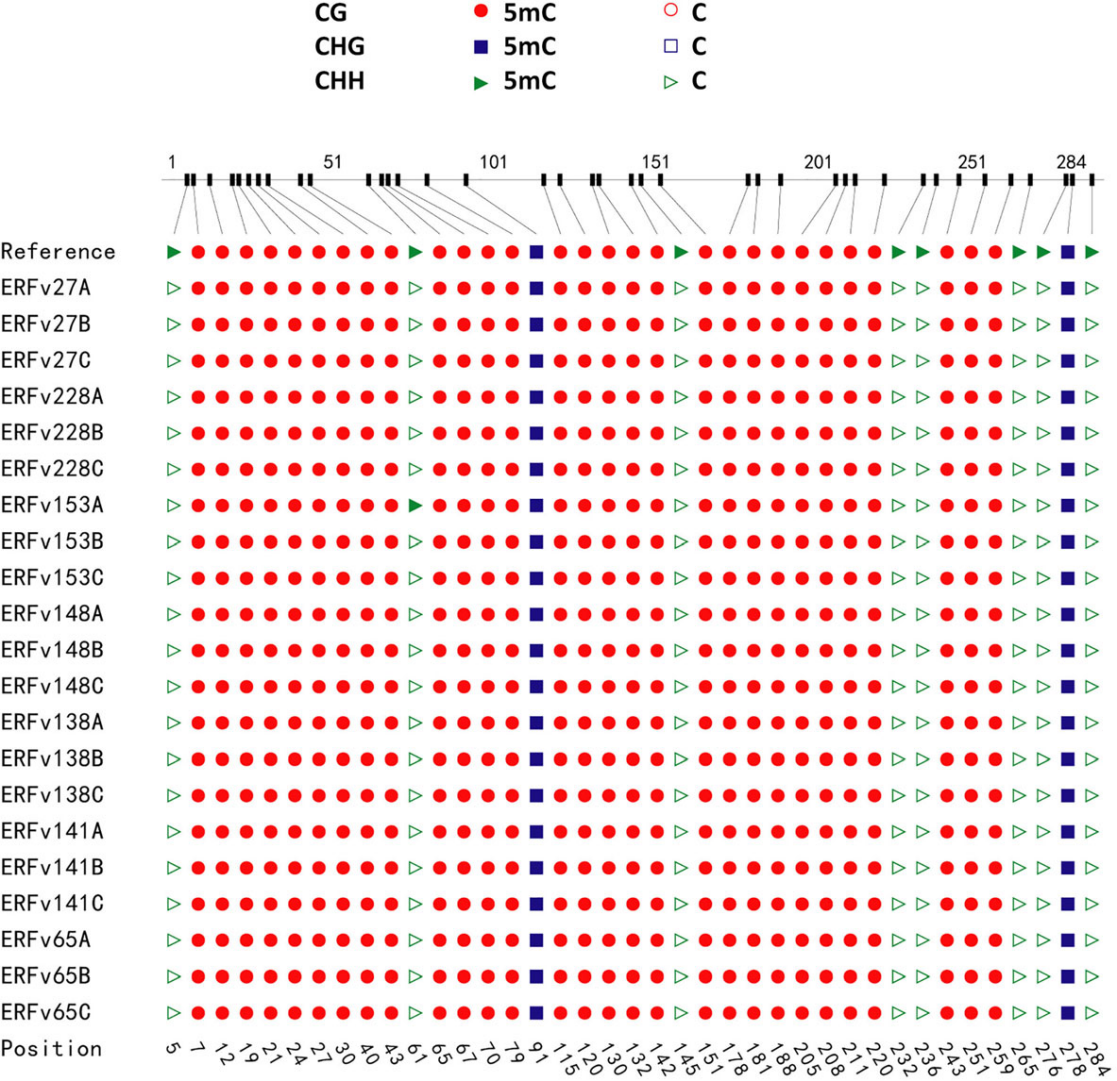
Methylation status of each cytosine in CpG enriched strawberry target region one. A total of 21 lines were assayed including control ERFv27, ERFv228, early flowering ERFv153, ERFv148, late flowering ERFv138, ERFv141, small rosette diameter ERFv65 respectively. A represents the parent generation, B and C represent the next generation progeny derived from A. The Class 1, Class 2, and Class 3 represent CG, CHG, and CHH methylation, respectively. Filled shapes indicate methylated cytosines, and open shapes indicate non methylated cytosines. The number along the bottom indicates the cytosine position in the sequences

Multivariate statistical analysis of the MSAP data indicated enrichment of methylation patterns within families. This clustering suggests that DNA methylation patterns are heritable and is in agreement with data from other analyses [65, 68, 69]. Despite this, the sampled MSAP loci are, at best, linked to the factors underlying the variation. Since they lack a genomic location they provide little utility beyond indicating that variation exists. In order to verify that DNA methylation patterns can be faithfully inherited from parents to offspring, methylation patterns were determined at defined loci. The loci were selected from an *in silico* analysis of the strawberry genome for enrichment in cytosine bases. These regions were randomly selected to assess the inheritance of methylation information and have no known bearing on the observed trait variation. To test the fidelity of inheritance, a total of 21 lines were assayed including the same parental lines examined by MSAP, namely, the early flowering lines ERFv153 and ERFv148; the late flowering lines ERFv138 and ERFv141; the small rosette diameter line ERFv65 (Fig. 6) and two control lines ERFv27, ERFv228 along with two siblings from the progeny of each selected parental line. Three target loci were selected from the *F. vesca* genome as being enriched for the presence of cytosine bases. The conversion efficiency of the sodium bisulfite treatment was adequate since each of cytosine bases were converted into uracil and sequenced as thymine when amplified from the Lambda genome spiked into each sample (Additional file 8: Figure S3a). The three target regions of the *F .vesca* genome sequenced after conversion with sodium bisulfite exhibited different levels of DNA methylation with 79 % of the cytosine bases methylated at target region one (Fig. 9), 59 % of the cytosine bases methylated at target region two (Additional file 8: Figure S3b), and no methylation observed at target region three (Additional file 8: Figure S3c). Faithful inheritance of cytosine methylation in all three sequence contexts (CG, CHG, and CHH) was observed in target regions one and three in all of the 21 lines tested (Fig. 9, Additional file 8: Figure S3c). At target region two, all methylated cytosine bases occurring in a CG context and the majority of methylated CHG and CHH (over 80 %) were faithfully inherited from parent to progeny. However, variation in methylation pattern was observed at sequence positions 277, 320, 337, 347 and 358 (Additional file 8: Figure S3b). Overall, these results indicated that DNA methylation is faithfully transmitted through meiosis and this appears to occur with greater fidelity at CG positions.

## Discussion

This study describes the generation of a novel hypomethylated population by treating seeds from a highly inbred *F. vesca* line with the cytidine analogue 5-azaC. We demonstrate the population contains expanded variation for a range of quantitative characters and that some of these novel phenotypes and methylation patterns are transmitted through meiosis. This resource likely contains novel epialleles and induced rearrangements whose identification can lead to a more complete understanding of the cellular mechanisms that determine the extent of quantitative trait variation. This population is an ideal resource to address questions surrounding the extent that DNA methylation patterns exert over quantitative trait variation. The function and utility of epigenetic variation has been the subject of debate and intensive research [70, 71]. Studies have demonstrated the effect of epigenetic alterations on phenotypic variation taking advantage of the resources available in *Arabidopsis*, where EpiRIL derived from *met1* mutants show increased trait variation [10]. The phenotypic variation observed among the EpiRIL points to the potential of epigenetic variation for rapidly inducing new variation [9, 10]. Currently, however, strawberry does not have similar mutants to develop such resources. The use of pharmacological agents, such as 5-azaC, that inhibit DNA methylation is an alternative method of developing such resources. The overall effect of this treatment is the generation of a population of plants that are genetically near-identical yet possess unique, hypomethylated DNA methylation patterns. The one major function of DNA methylation is to silence repetitive elements although cytosine methylation occurs along the length of annotated genes [72, 73]. Also, alterations in DNA methylation hold the potential to change the regulation over transcription, altering the magnitude of gene expression and generating novel phenotypic variation.

Attributing phenotypic variation to epigenetic polymorphism can be confounded by the presence of genetic variation segregating in a population. Disentangling epigenetic and genetic contributions is challenging. To minimize the influence of genetic variation, the hypomethylated *F. vesca* population described in this study was developed using a highly inbred genotype of Hawaii 4. Inbreeding through single-seed descent for eight generations would have resulted in >99 % of the heterozygous alleles in the original Hawaii 4 ecotype to become homozygous. The high frequency of homozygous alleles was confirmed by the AFLP marker analysis, which revealed no polymorphism. AFLP markers can simultaneously assay large numbers of loci throughout the genome without any prior sequence information [67]. The results from this analysis and the history of the inbred *F. vesca* material indicated that the H4S8 population is genetically uniform. The detection of identical alleles at 219 loci across the seven chromosomes supports the premise that only *F. vesca* alleles derived from Hawaii 4 were present indicating that no alien *F. vesca* alleles had been introduced into the population through inadvertent hybridization. However, the possibility remained that SNP variation might be present among the hypomethylated lines and this could represent a significant source of variation should 5-azaC act as a mutagen in addition to reducing DNA methylation. Since the material used in this study directly descended from the material used to generate the *F. vesca* genome sequence, the opportunity existed to estimate the potential of 5-azaC to behave as a mutagen and induce SNP variation. The relationship between the *F. vesca* material used in this study (H4S8) and the material used to generate the reference genome (H4S4) was an additional four generations of inbreeding. Whole genome sequence data were generated from seven individuals, four of which were exposed to high concentrations of 5-azaC (50 mM) and three from control lines (0 mM). These data were aligned to the reference genome sequence and SNP identified. The difference between the reference genome (H4S4) and the untreated H4S8 material indicates the level of SNP due to spontaneous changes. Polymorphisms between the reference genome and the 5-azaC treated individuals would include the same spontaneous mutation rate as the control lines in addition to any mutagenic effects of 5-azaC.

It is challenging to reliably identify loci with single nucleotide polymorphisms from alignments of short read sequence data to a draft reference genome sequence. In addition to obtaining adequate coverage of the genome, sufficient sequencing depth is required to discriminate actual polymorphisms from those generated by artifacts including short read sequencing and alignment errors that are compounded by errors inherent in the reference genome assembly. Using parameters to detect high quality SNP, 148 loci were detected as possessing an allele that might have occurred spontaneously. This number increased slightly to 153 loci when the 5-azaC lines were included, providing no evidence for appreciable mutagenic activity from 5-azaC. This experiment assesses the potency of 5-azaC to induce new SNP and is not intended as an estimate of the spontaneous mutation rate. The SNP data emphasize the similarity among the control material and lines treated with the highest concentrations of 5-azaC. A total of 1548 loci were detected with SNP revealing polymorphism among the control and 5-azaC treated lines, a number that was considerably less than the number of loci where the identical genotype was found in (29,137) the control and 5-azaC treated lines yet differed to the reference allele. These data suggest that the individual line selected for sequencing was heterozygous at these loci or perhaps that the sequenced material was derived from more than a single individual. The SNP data indicate that the H4S8 material is heterozygous at a minimum of 1208 loci and is homozygous for alleles exclusive to the 5-azaC treated lines at 340 loci. Sequencing of additional control material might reduce this number. Nevertheless, 183 of these loci were found in annotated genes with 11 predicted to introduce a premiture stop codon or alter splicing and 119 to change the amino acid sequence of the translated protein. The genome sequencing data verified the near-identical genetic background of the *F. vesca* control and 5-azaC treated populations. Our observation is consistent with a previous analysis demonstrating that 5-azaC lacks signficant activity to change the primary DNA sequence and functions as an efficient inhibitor of DNA methylation [74]. However, these data do not rule out the presence of genetic polymorphisms from the 5-azaC population, as hypomethylation might result in an increase in transposition activity or other chromosomal rearrangements that are difficult to detect from short read alignment data to draft genome assemblies. Yet the absence of fragment length polymorphisms in the AFLP data suggest that these events do not occur at a high frequency. The detection of any underlying alleles controlling the expanded trait variation will require segregation analysis to identify their origin and location. Only then will the underlying alleles be revealed as being either genetic or epigenetic in nature.

As anticipated, the major effect of exposure to 5-azaC was the induction of DNA methylation changes and these were detected in the hypomethylated population through analysis using MSAP markers. The MSAP data revealed that the genome of *F. vesca* appears to be predominantly unmethylated with 62 % of the sampled cytosines lacking a methyl group. The estimation of hemimethylation among cytosine bases in *F. vesca* was placed at 8 % (type II / (type II + type III + type IV), Table 2). This figure is comparable to estimations made in related species such as rose (10 %) and apple (6 %) [75, 76]. In our study 54 % (type III / (type II + type III + type IV), Table 2) of methylation occurs at the internal cytosine on each strand and 38 % (type IV / (type II + type III + type IV), Table 2) were on all four cytosine bases, while the opposite was observed in rose and apple, in which the majority (55 and 70 % respectively) of methylation occurred on all four cytosine bases and lower frequencies (35 and 24 % respectively) on the internal cytosine of each strand. Although this analysis provides some understanding of the *F. vesca* epigenome, the MSAP data lack positional information complicating their interpretation and rendering them largely descriptive. Further, the MSAP analysis is restricted to examining methylation status only at CCGG sites, as such these estimates are made using a subset of the potential CG loci. Thus, these estimates might contain some bias. Nevertheless, we utilized the ability of MSAP to rapidly determine the level of DNA methylation and showed that plants exposed to 5-azaC had a reduction in the level of methylation at the sampled loci. These data provide the evidence that the 5-azaC treatment induced epigenetic variation resulting in the development of a *F. vesca* hypomethylated population. As anticipated, methylation depletion followed a dose response where greater depletion was correlated with a higher 5-azaC concentration. The overall trend observed among the methylation classes described a shift in the frequency of type IV to type III and type I classes with the 5-azaC concentration increasing. However, only at the highest 5-azaC concentration did the frequencies change appreciably. Nevertheless, inhibition of DNA methylation by 5azaC is expected to generate novel differentially methylated regions (DMR) when compared to control plants, some of which might affect transcriptional activity leading to increased phenotypic variation.

Moderate dysregulation of gene expression can be exploited to alter quantitative characters. The quantitative traits that were focused on in this study included flowering time and rosette diameter, although other morphological and physiological changes were observed. Similar to the results obtained from the analysis of *Arabidopsis* EpiRILs [9] and a range of studies in other species using 5-azaC [43, 45, 77], we observed a broader range of quantitative phenotypic variation among the hypomethylated lines compared to the control population. The expanded variation observed for flowering time yielded plants of early and late flowering at both tails of the distribution (Fig. 3a). However, for rosette diameter only plants with a smaller rosette were observed (Fig. 3b), perhaps reflecting a lower growth rate. Similar data have been observed by others where dwarfed seedlings were found in rice [43]. In both *Arabidopsis* and *Brassica rapa*, plants tended to flower late after exposure to 5-azaC [77, 78], while in both flax and potato, flowering time variants following 5-azaC treatment reached anthesis significantly earlier than controls [48, 79]. The early flowering strawberry lines identified in this study did not appear stunted in their growth habit as was observed in the flax early flowering variants, suggesting that these phenotypes are not merely the result of stress.

Transmission of the phenotypic variants through meiosis suggests that the phenotypic changes are due to heritable factors rather than merely resulting from stress due to 5azaC exposure. This was exemplified by the progeny from the late flowering variant ERFv141 where >95 % of the lines flowered significantly later than the control population mean. It was also observed that the early flowering trait was transmitted to the following generation although the distributions were less distinct than the ERFv141 material. These results contrasted with the transmission of small rosette diameter from ERFv65, which was not observed to be significantly different from control although small variants were observed among the progeny. While the underlying factors controlling the flowering time variation remain undetermined, future characterization of these lines could uncover the factors responsible. MSAP is ill suited for identifying the underlying factors, due to the low number of sites sampled, restriction site distribution and the low resolution of methylation information provided. However, accessibility of this technology makes it ideal for initial characterization and assessment of variation in methylation patterns. Analysis of the H4S9 progeny derived from the selected lines possessing early flowering, late flowering, small rosette and control phenotypes revealed different DNA methylation patterns. The progeny demonstrating inheritance of variant flowering time were derived from two independent lines for both early and late flowering behavior. This contrasted with the progeny for small rosette diameter that was derived from ERFv65 only. Clustering of MSAP patterns from phenotypically related individuals for flowering time suggests that some loci might be associated with the factors controlling these characters. Whereas the clustering of MSAP data in progeny from ERFv65 is more likely restricted to line specific methylation changes, despite the smallest variants being analyzed. The distances separating these clusters were demonstrated to be statistically significant. This was especially pronounced for the early flowering phenotype. The early flowering line ERFv153, which was treated with 50 mM 5-azaC, was the most hypomethylated with the methylation level at sampled loci reduced by 19.1 %. Clustering of the H4S9 generation by phenotype (Fig. 9) contrasts with the unrelated pattern observed among the individuals of H4S8 generation (Fig. 3) where the changes occurred in all directions in each dimension of the data summarized using eigenvalues. These data suggest that some of the DNA methylation patterns might be associated, possibly through linkage, with underlying heritable factors but are themselves unlikely to be directly related.

Comparing the inheritance of DNA methylation patterns using MSAP data is complicated as fragments are resolved on different acrylamide gels under differing electrophoresis conditions. To overcome these limitations, DNA methylation patterns at three different target loci were amplified after conversion using sodium bisulfite. This approach ensured direct comparison of the methylation status of the same cytosine bases from 21 lines from which the fidelity of inheritance was estimated from the transmission of marks from each of the seven parents to two progeny lines. The three target regions are unrelated to any trait, but the methylation patterns are faithfully inherited for majority of the cytosine bases analyzed. Inheritance was found in target region one and three in each sequence context. Inheritance was observed at CG sites in target region two and only slight variation was observed in CHG and CHH contexts. This variation might result from ncRNA-directed *de novo* methylation, sequencing errors or polymorphism to the reference genome sequence. Nevertheless, these data indicate faithful transmission of DNA methylation patterns from parent to progeny at the majority of methylation sites particularly CG sites.

Flowering time is an important multigenic trait commonly targeted in plant breeding programs. In a situation where the introgression of alleles from wild species can compromise quality traits, the use of compounds such as 5-azaC to induce desirable novel variation without changing the combination of alleles selected by breeders could play a part in future crop improvement strategies. This study establishes a resource containing expanded phenotypic changes, which were transmitted through meiosis and can potentially be subjected to selection, demonstrating the potential of this approach. Although this resource was established in highly inbred material and whole genome DNA sequence analysis indicated that exposure to 5-azaC did not induce new SNP, genetic variation such as chromosomal rearrangements might have resulted from the induced hypomethylation. However, the frequency of their occurrence is not high since no fragment length variation was observed using AFLP. Further research is required to identify the factors, underlying these phenotypic traits. The material described makes an ideal resource to examine the heritability of this variation through multiple generations and upon the identification of the underlying factors, and the potential of drugs such as 5-azaC for generating useful variation.

## Conclusions

This study describes the generation of a population exhibiting expanded variation in strawberry. *F. vesca* is increasingly being used as a model plant for the Rosaceae family, and its clonal and sexual strategies offer new experimental opportunities to evaluate the transmission of DNA methylation marks. We confirmed that the Hawaii 4 material used for population development was genetically uniform and that no appreciable increase in SNP frequency was induced by exposure to 5aza-C. Increased variation in DNA methylation profiles and phenotypic variation for several quantitative traits was observed. Moreover, the value of this resource was increased with the demonstration that the variant traits and DNA methylation patterns could be transmitted through meiosis. However, further work and future material development is required to establish associations between the trait and its underlying molecular variation.

## Additional files

Additional file 1: Table S1. Adaptors and primers used in AFLP and MSAP analysis. **a.** Sequences of adaptors and primers used for pre-selective amplification and selective amplification in AFLP; **b.** Sequences of adaptors and primers used for pre-selective amplification and selective amplification in MSAP. (DOC 59 kb)
Additional file 2: Figure S1.
*Hpa*II (H), *Msp*I (M) sensitivity to methylation at CCGG sites and scoring of MS-AFLP bands. The type IV band represents full methylation, we added the fully methylated 5′mCCGG sequence here. The “1” represents the presence of bands and “0” represents the absence of bands for scoring purposes. (PNG 69 kb)
Additional file 3: Table S2. Primers used in bisulfite sequencing PCR. (DOC 47 kb)
Additional file 4: Figure S2. Example of AFLP electrophoretic patterns in control lines and epi-mutant lines. Banding pattern was generated using theprimer combination *Eco*RI+ AC and *Mse*I+ CAA C1-C5: five control lines. 1–22: randomly selected 22 hypomethylated lines. (PNG 545 kb)
Additional file 5: Table S3. Summary of the depth and breath of sequence reads alignements in the control and 5-azaC treatment lines. **a.** The distribution of coverage in each line across the genome; **b.** The distribution of depth of coverage in each line across the genome. (DOC 61 kb)
Additional file 6: Table S4. Analysis of variance (ANOVA) comparing cytosine methylation levels among exposure to different concentration of 5-azaC. (DOC 55 kb)
Additional file 7: Table S5. A summary of DNA methylation profile in five control lines and 22 randomly selected epimutagenized population lines. (DOC 77 kb)
Additional file 8: Figure S3. Methylation status of each cytosine in two CpG enriched strawberry target regions and the unmethylated Lambda control. Assessment of bisulfite conversion efficiency using Lambda as an unmethylated control (**a**), target region two (**b**), and target region three (**c**). A total of 21 lines were assayedincluding control ERFv27, ERFv228, early flowering ERFv 153, ERFv 148, late flowering ERFv 138, ERFv 141, small rosette diameter ERFv 65 respectively. A represents the parent generation, B and C represent the next generation progeny derived from A. The Class 1, Class 2, and Class 3 represent CG, CHG, and CHH methylation, respectively. Filled shapes indicate methylated cytosines, and open shapes indicate non methylated cytosines. The number along the bottom indicates the cytosine position in the sequences. (JPG 750 kb)

## Abbreviations

5-azaC: 5-azacytidine
AFLP: Amplified Fragment Length Polymorphism
AMOVA: Analysis of Molecular Variance
DMR: Differentially Methylated Regions
EpiRILs: Epigenetic Recombinant Inbred Lines
GATK: Genome Analysis Toolkit
MSAP: Methylation Sensitive Amplified Polymorphisms
PCoA: Principal Coordinates Analysis
SNP: Single Nucleotide Polymorphism
