# Supplementary material for: Quantitative trait variation is revealed in a novel hypomethylated population of woodland strawberry (Fragaria vesca)
Source: BMC Plant Biol. 2016 Nov 4;16:240. doi: 10.1186/s12870-016-0936-8 (PMC5095969; doi:10.1186/s12870-016-0936-8)
Supplement: Additional file 1: Table S1. — Adaptors and primers used in AFLP and MSAP analysis. a. Sequences of adaptors and primers used for pre-selective amplification and selective amplification in AFLP; b. Sequences of adaptors and primers used for pre-selective amplification and selective amplification in MSAP. (DOC 59 kb) [file 12870_2016_936_MOESM1_ESM.doc]

**Additional file 1: Table S1** Adaptors and primers used in AFLP and MSAP analysis

**a.** Sequences of adaptors and primers used for pre-selective amplification and

selective amplification in AFLP

| Adapters/Primers | *Eco*RI | *Mse*I |
| --- | --- | --- |
|
| Adapter | 5-CTCGTAGACTGCGTACC-3 | 5-GACGATGAGTCCTGAG-3 |
|  | 3-CATCTGACGCATGGTTAA-5 | 3-TACTCAGGACTCAT-5 |
| Pre-amplification primers | 5-GACTGCGTACCAATTC A-3 | 5-GATGAGTCCTGAGTAA C-3 |
| Selective primers | 5-GACTGCGTACCAATTC AC-3 | 5-GATGAGTCCTGAGTAA CAA-3 |
|  | 5-GACTGCGTACCAATTC AC-3 | 5-GATGAGTCCTGAGTAA CAT-3 |
|  | 5-GACTGCGTACCAATTC AC-3 | 5-GATGAGTCCTGAGTAA CCA-3 |
|  | 5-GACTGCGTACCAATTC AC-3 | 5-GATGAGTCCTGAGTAA CGT-3 |

**b.** Sequences of adaptors and primers used for pre-selective amplification and selective amplification in MSAP

| Adapters/primers | *Eco*RI | *Hpa*II/*Msp*I |
| --- | --- | --- |
| Adapter | 5-CTCGTAGACTGCGTACC-3 | 5-GACGATGAGTCTAGAA-3 |
|  | 3-CATCTGACGCATGGTTAA-5 | 3-CTACTCAGATCTTGC-5 |
| Pre-amplification primers | 5-GACTGCGTACCAATTC A-3 | 5-GATGAGTCTAGAACGG T-3 |
| Selective primers | 5-GACTGCGTACCAATTC ACT-3 | 5-GATGAGTCTAGAACGG TAA-3 |
|  | 5-GACTGCGTACCAATTC ACT-3 | 5-GATGAGTCTAGAACGG TTA-3 |
|  | 5-GACTGCGTACCAATTC ACT-3 | 5-GATGAGTCTAGAACGG TCA-3 |
|  | 5-GACTGCGTACCAATTC ACT-3 | 5-GATGAGTCTAGAACGG TGA-3 |
|  | 5-GACTGCGTACCAATTC ACT-3 | 5-GATGAGTCTAGAACGG TTC-3 |
|  | 5-GACTGCGTACCAATTC ACT-3 | 5-GATGAGTCTAGAACGG TTG-3 |
|  | 5-GACTGCGTACCAATTC ACT-3 | 5-GATGAGTCTAGAACGG TTT-3 |
|  | 5-GACTGCGTACCAATTC ACA-3 | 5-GATGAGTCTAGAACGG TAA-3 |
|  | 5-GACTGCGTACCAATTC ACA-3 | 5-GATGAGTCTAGAACGG TTA-3 |
|  | 5-GACTGCGTACCAATTC ACA-3 | 5-GATGAGTCTAGAACGG TTT-3 |
