# Supplementary figures and images for: Quantitative trait variation is revealed in a novel hypomethylated population of woodland strawberry (Fragaria vesca)
Source: BMC Plant Biol. 2016 Nov 4;16:240. doi: 10.1186/s12870-016-0936-8 (PMC5095969; doi:10.1186/s12870-016-0936-8)

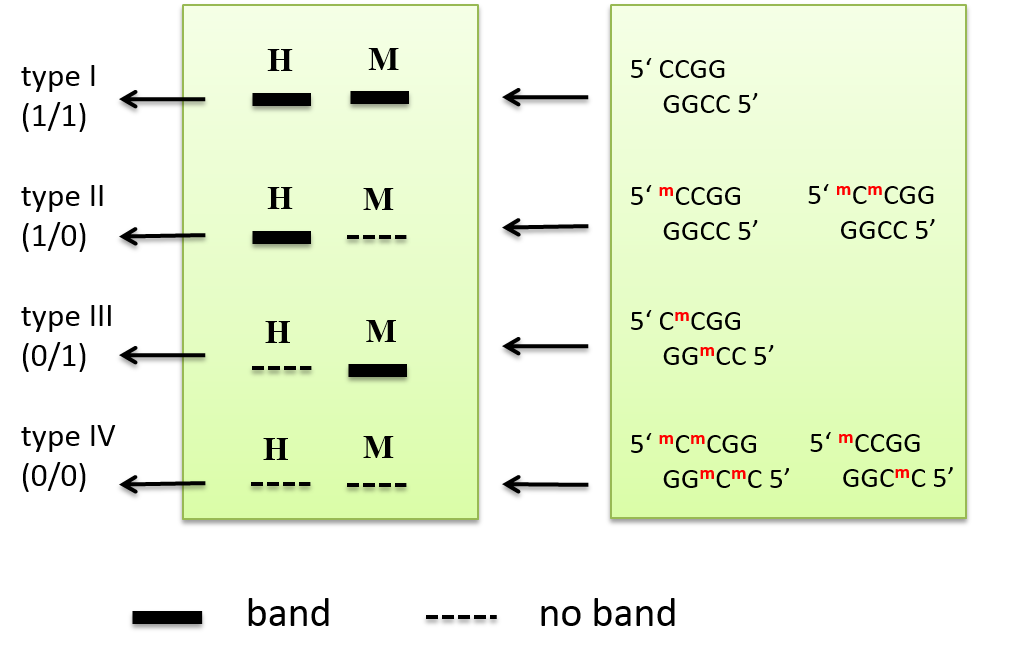

Supplement: Additional file 2: Figure S1. — HpaII (H), MspI (M) sensitivity to methylation at CCGG sites and scoring of MS-AFLP bands. The type IV band represents full methylation, we added the fully methylated 5′mCCGG sequence here. The “1” represents the presence of bands and “0” represents the absence of bands for scoring purposes. (PNG 69 kb) [file 12870_2016_936_MOESM2_ESM.png]

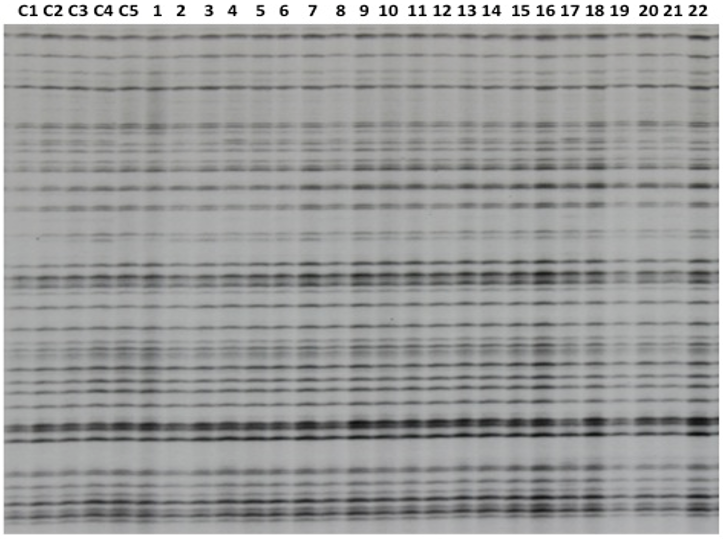

Supplement: Additional file 4: Figure S2. — Example of AFLP electrophoretic patterns in control lines and epi-mutant lines. Banding pattern was generated using theprimer combination EcoRI+ AC and MseI+ CAA C1-C5: five control lines. 1–22: randomly selected 22 hypomethylated lines. (PNG 545 kb) [file 12870_2016_936_MOESM4_ESM.png]

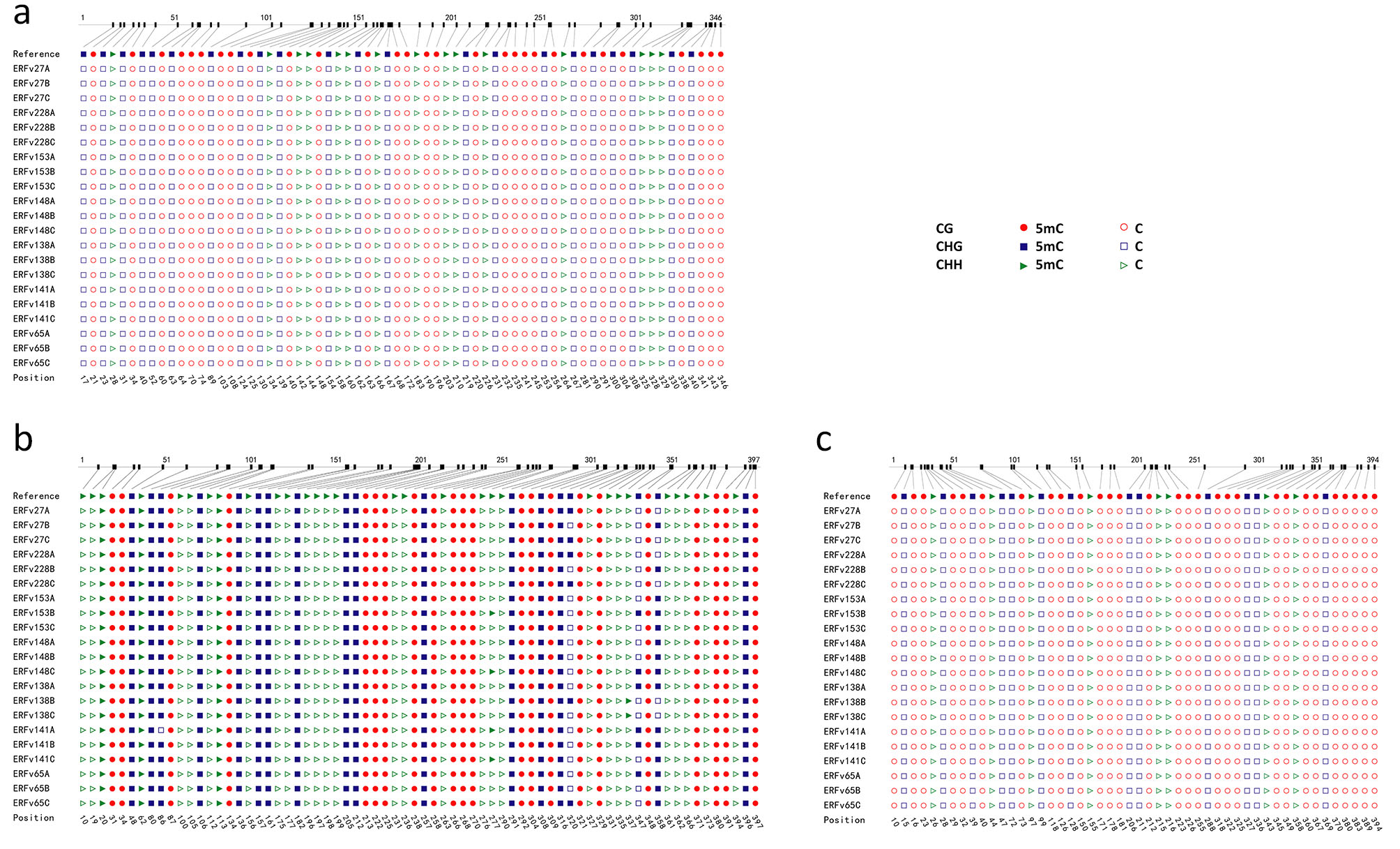

Supplement: Additional file 8: Figure S3. — Methylation status of each cytosine in two CpG enriched strawberry target regions and the unmethylated Lambda control. Assessment of bisulfite conversion efficiency using Lambda as an unmethylated control (a), target region two (b), and target region three (c). A total of 21 lines were assayedincluding control ERFv27, ERFv228, early flowering ERFv 153, ERFv 148, late flowering ERFv 138, ERFv 141, small rosette diameter ERFv 65 respectively. A represents the parent generation, B and C represent the next generation progeny derived from A. The Class 1, Class 2, and Class 3 represent CG, CHG, and CHH methylation, respectively. Filled shapes indicate methylated cytosines, and open shapes indicate non methylated cytosines. The number along the bottom indicates the cytosine position in the sequences. (JPG 750 kb) [file 12870_2016_936_MOESM8_ESM.jpg]
