# Supplementary material for: Quantitative trait variation is revealed in a novel hypomethylated population of woodland strawberry (Fragaria vesca)
Source: BMC Plant Biol. 2016 Nov 4;16:240. doi: 10.1186/s12870-016-0936-8 (PMC5095969; doi:10.1186/s12870-016-0936-8)
Supplement: Additional file 3: Table S2. — Primers used in bisulfite sequencing PCR. (DOC 47 kb) [file 12870_2016_936_MOESM3_ESM.doc]

**Additional file 3: Table S2** Primers used in bisulfite sequencing PCR

|  | Forward Primer Sequence | Reverse Primer Sequence |
| --- | --- | --- |
| Target region one | 5-GAGTAGAGGGTGGTTGATT-3 | 5-TCTCACCAACCAATCAAATC-3 |
| Target region two | 5-GAGGATGATATTGTGAGGTTA-3 | 5-CTACACCCTATAAACACCTA-3 |
| Target region three | 5-TTGAGTAGAAGTGAATGAG-3 | 5-AACCTCCAAACTACTCTCTA-3 |
| Lambda primer | 5-GATGTGTAGGTTATGGTGA-3 | 5-CCATCACACTCACATAAC-3 |
