# Supplementary material for: Quantitative trait variation is revealed in a novel hypomethylated population of woodland strawberry (Fragaria vesca)
Source: BMC Plant Biol. 2016 Nov 4;16:240. doi: 10.1186/s12870-016-0936-8 (PMC5095969; doi:10.1186/s12870-016-0936-8)
Supplement: Additional file 5: Table S3. — Summary of the depth and breath of sequence reads alignements in the control and 5-azaC treatment lines. a. The distribution of coverage in each line across the genome; b. The distribution of depth of coverage in each line across the genome. (DOC 61 kb) [file 12870_2016_936_MOESM5_ESM.doc]

**Additional file 5: Table S3** Summary of the depth and breath of sequence reads alignements in the control and 5-azaC treatment lines

**a. The distribution of coverage in each line across the genome**

|  | Reference | Con 1 | Con 2 | Con 3 | ERFv 140 | ERFv 246 | ERFv 132 | ERFv 153 |
| --- | --- | --- | --- | --- | --- | --- | --- | --- |
| LG1 | 24178039 | 21317245 | 21350972 | 21317109 | 21331069 | 21361619 | 21366204 | 21348926 |
| LG2 | 35504443 | 31197786 | 31248980 | 31199229 | 31222325 | 31265870 | 31275762 | 31246727 |
| LG3 | 29376571 | 26342781 | 26385557 | 26345840 | 26362986 | 26400600 | 26407963 | 26383537 |
| LG4 | 24141177 | 21802964 | 21840725 | 21805867 | 21817819 | 21851261 | 21857516 | 21837786 |
| LG5 | 30775793 | 27706440 | 27756275 | 27712527 | 27728668 | 27769668 | 27777504 | 27749944 |
| LG6 | 39918795 | 35990843 | 36052431 | 35998091 | 36022429 | 36072557 | 36080868 | 36049501 |
| LG7 | 24052591 | 22234862 | 22271854 | 22239518 | 22253469 | 22281798 | 22285625 | 22266143 |
| All | 207947409 | 186592921 | 186906794 | 186618181 | 186738765 | 187003373 | 187051442 | 186882564 |

**b. The distribution of depth of coverage in each line across the genome**

|  | Con 1 | Con 2 | Con 3 | ERFv 140 | ERFv 246 | ERFv 132 | ERFv 153 |
| --- | --- | --- | --- | --- | --- | --- | --- |
| LG1 | 7.94 | 10.5 | 7.8 | 8.72 | 11.73 | 13.69 | 9.46 |
| LG2 | 7.7 | 10.2 | 7.6 | 8.47 | 11.48 | 13.32 | 9.34 |
| LG3 | 8.22 | 11.03 | 8.14 | 9.13 | 12.24 | 14.37 | 9.85 |
| LG4 | 8.01 | 10.8 | 8.03 | 8.95 | 12.24 | 14.09 | 9.93 |
| LG5 | 8.94 | 10.7 | 7.92 | 8.89 | 11.97 | 14.01 | 9.66 |
| LG6 | 8.22 | 12.06 | 8.9 | 9.92 | 13.45 | 15.62 | 10.6 |
| LG7 | 8.25 | 10.96 | 8.1 | 9.08 | 12.25 | 14.28 | 9.95 |
| Average | 8.18 | 10.89 | 8.07 | 9.02 | 12.19 | 14.20 | 9.83 |
|  | | | | | | | |
