# Supplementary material for: Quantitative trait variation is revealed in a novel hypomethylated population of woodland strawberry (Fragaria vesca)
Source: BMC Plant Biol. 2016 Nov 4;16:240. doi: 10.1186/s12870-016-0936-8 (PMC5095969; doi:10.1186/s12870-016-0936-8)
Supplement: Additional file 6: Table S4. — Analysis of variance (ANOVA) comparing cytosine methylation levels among exposure to different concentration of 5-azaC. (DOC 55 kb) [file 12870_2016_936_MOESM6_ESM.doc]

**Additional file 6: Table S4** Analysis of variance (ANOVA) comparing cytosine methylation levels among exposure to different concentration of 5-azaC

Summary of analysis of variance

|  | Df | Sum Sq | Mean Sq | F value | P (>F) |
| --- | --- | --- | --- | --- | --- |
| Treatment | 4 | 139.03 | 34.758 | 3.2442 | 0.03097 * |
| Residuals | 22 | 235.7 | 10.714 |  |  |

Tukey multiple comparisons of means (95% family-wise confidence level)

| Treatment | diff | lwr | upr | adj |
| --- | --- | --- | --- | --- |
| 1.0mM-0mM | -1.708 | -7.8501 | 4.43405 | 0.92 |
| 5.0mM-0mM | -1.179 | -7.6936 | 5.33563 | 0.98242 |
| 20.0mM-0mM | -3.2397 | -8.9262 | 2.44672 | 0.45978 |
| 50.0mM-0mM | -6.4973 | -12.378 | -0.6168 | 0.02557* |
| 5.0mM-1.0mM | 0.529 | -5.9856 | 7.04363 | 0.99919 |
| 20.0mM-1.0mM | -1.5317 | -7.2182 | 4.15472 | 0.92806 |
| 50.0mM-1.0mM | -4.7893 | -10.67 | 1.09123 | 0.14815 |
| 20.0mM-5.0mM | -2.0607 | -8.1477 | 4.02625 | 0.85053 |
| 50.0mM-5.0mM | -5.3183 | -11.587 | 0.95037 | 0.12289 |
| 50.0mM-20.0mM | -3.2576 | -8.6606 | 2.14533 | 0.40455 |
| * p < 0.05 |  |  |  |  |
