# Supplementary material for: Quantitative trait variation is revealed in a novel hypomethylated population of woodland strawberry (Fragaria vesca)
Source: BMC Plant Biol. 2016 Nov 4;16:240. doi: 10.1186/s12870-016-0936-8 (PMC5095969; doi:10.1186/s12870-016-0936-8)
Supplement: Additional file 7: Table S5. — A summary of DNA methylation profile in five control lines and 22 randomly selected epimutagenized population lines. (DOC 77 kb) [file 12870_2016_936_MOESM7_ESM.doc]

**Additional file 7: Table S5** A summary of DNA methylation profile in five control lines and 22 randomly selected epimutagenized population lines

| Number on gel | Lines | Treatment (mM) | Type I (1/1) | Type II (1/0) | Type III (0/1) | Type IV (0/0) | Methylated cytosine | Relative DNA methylation % |
| --- | --- | --- | --- | --- | --- | --- | --- | --- |
|  |  |  |  |  |  |  | % |  |
| 1 | Control 1 | 0 | 114 | 6 | 73 | 53 | 37.6 | -0.77 |
| 2 | Control 2 | 0 | 104 | 15 | 77 | 50 | 39.02 | 0.65 |
| 3 | Control 3 | 0 | 108 | 10 | 70 | 58 | 39.84 | 1.46 |
| 4 | Control 4 | 0 | 112 | 16 | 69 | 49 | 37.2 | -1.18 |
| 5 | Control 5 | 0 | 110 | 10 | 74 | 52 | 38.21 | -0.16 |
| 6 | ERFv 11 | 1 | 110 | 19 | 71 | 46 | 36.99 | -1.38 |
| 7 | ERFv 16 | 1 | 111 | 13 | 72 | 50 | 37.6 | -0.77 |
| 8 | ERFv 168 | 1 | 110 | 7 | 84 | 45 | 36.79 | -1.59 |
| 9 | ERFv 259 | 1 | 110 | 7 | 84 | 45 | 36.79 | -1.59 |
| 10 | ERFv 312 | 1 | 117 | 7 | 78 | 44 | 35.16 | -3.21 |
| 11 | ERFv 134 | 5 | 116 | 9 | 75 | 46 | 35.77 | -2.6 |
| 12 | ERFv 137 | 5 | 111 | 9 | 68 | 58 | 39.23 | 0.85 |
| 13 | ERFv 157 | 5 | 114 | 10 | 75 | 47 | 36.38 | -1.99 |
| 14 | ERFv 345 | 5 | 113 | 6 | 76 | 51 | 37.4 | -0.98 |
| 15 | ERFv 45 | 20 | 116 | 12 | 63 | 55 | 37.6 | -0.77 |
| 16 | ERFv 141 | 20 | 115 | 15 | 74 | 42 | 35.16 | -3.21 |
| 17 | ERFv 147 | 20 | 118 | 10 | 77 | 41 | 34.35 | -4.02 |
| 18 | ERFv 217 | 20 | 111 | 6 | 92 | 37 | 34.96 | -3.41 |
| 19 | ERFv 295 | 20 | 122 | 5 | 75 | 44 | 34.15 | -4.23 |
| 20 | ERFv 328 | 20 | 120 | 6 | 78 | 42 | 34.15 | -4.23 |
| 21 | ERFv 329 | 20 | 121 | 12 | 63 | 50 | 35.57 | -2.8 |
| 22 | ERFv 95 | 50 | 123 | 11 | 71 | 41 | 33.33 | -5.04 |
| 23 | ERFv 127 | 50 | 120 | 12 | 73 | 41 | 33.94 | -4.43 |
| 24 | ERFv 132 | 50 | 113 | 14 | 78 | 41 | 35.37 | -3.01 |
| 25 | ERFv 140 | 50 | 111 | 10 | 76 | 49 | 37.4 | -0.98 |
| 26 | ERFv 153 | 50 | 165 | 8 | 60 | 13 | 19.11 | -19.27 |
| 27 | ERFv 246 | 50 | 126 | 12 | 70 | 38 | 32.11 | -6.26 |
| NA | Control lines mean | NA | 110 | 11 | 73 | 52 | 38.37 | 0 |
| NA | Epimutagenized lines mean | NA | 118 | 10 | 74 | 44 | 34.96 | -3.41 |

Methylated cytosine(%) = [(II*2 + III*2 + IV*4) / ((I + II + III + IV)*4)]* 100

RelativeDNA methylation (%) = Methylated cytosine (%) in treatment lines - Methylated cytosine (%) in control lines.

A total of 246 amplified loci were scored in every individual.
